# Factors Influencing the Intra-Oral Movement of the Bit: A Cadaveric Study †

**DOI:** 10.3390/ani15182648

**Published:** 2025-09-10

**Authors:** Elke Pollaris, Chris Hannes, Maarten Haspeslagh, Wouter Demey, Stijn Teysen, Bernard Boussauw, Lieven Vlaminck

**Affiliations:** 1 Equine Clinic De Morette by Equine Care Group, 1730 Asse, Belgium; 2 Independent Researcher, 2290 Vorselaar, Belgium; 3 Department of Surgery and Anaesthesiology of Domestic Animals, Faculty of Veterinary Medicine, Ghent University, 9820 Merelbeke, Belgium; 4 Independent Researcher, 3290 Schaffen, Belgium; 5 Independent Researcher, 1730 Asse, Belgium; 6 Equine Clinic De Bosdreef by Equine Care Group, 9880 Lokeren, Belgium

**Keywords:** bit-related trauma, intra-oral bit movement, equestrian sport, rein tension, rein angle, bit design, bit size

## Abstract

Bit-related trauma is a welfare issue in equestrian sports; therefore, a proper understanding of how the bit behaves inside the mouth is of importance. The objective of this study is to investigate the influence of bit design (snaffle bits/single/double-jointed; ring/eggbutt and curb bits), size, rein tension, and rein angle on the intra-oral movement of the bit in ex vivo specimens. The lateral and posterior movement of different bits was measured on cadaver heads under different circumstances. This ex vivo model, while not fully replicating natural conditions, provided insight into potential bit-related movements. Lateral displacement of the snaffle bit was significantly greater with higher unilateral rein tension. Bits that were too wide exhibited significantly more lateral and posterior displacement under unilateral rein tension. Posterior displacement increased with both unilateral and bilateral rein tensions. Using a bit that is too wide, using reins at a 20° angle, and applying 4 kg of rein tension were identified as risk factors for contact with the mesial gingival tissues of the mandibular 06s. Similar patterns were observed with curb bits, although bit movement was more restricted, and no contact with the mesial gingival tissues of the mandibular 06s was noted. This study demonstrated that bit design, bit size, rein angle, and rein tension all influence bit movement in the horse’s mouth.

## Simple Summary

This study looked at how different factors affect the range of motion of a bit inside a horse’s mouth. Horse cadaver heads were used to test various bit designs and sizes under different rein tensions and angles. It was measured how far the bit moved sideways (laterally) and backward (posteriorly) when reins were pulled in different ways. The results showed that snaffle bits moved more when higher rein tension was applied, especially at a 20° angle and with unilateral (one-sided) rein use. Bits that were too wide for the horse’s head moved more in both (lateral and posterior) directions and were more likely to come into contact directly with the first cheek tooth in the lower jaw. Although curb bits showed less movement overall, similar patterns were observed. The study suggests that using the correct bit size and managing rein tension could help reduce bit movement inside the horse’s mouth. However, the results are limited because the study used cadaver heads, which lack natural muscle tension from the tongue and cheeks. Also, not all bit types were tested.

## 1. Introduction

The bit is a traditional piece of equestrian equipment placed in the horse’s mouth, resting in the interdental space (the gap between incisors and premolars). It functions primarily as a means of communication and control, transmitting subtle cues from the rider through pressure on various oral structures such as the tongue, bars, and lips, depending on bit design and rein interaction [1].

Bit-related trauma is a welfare issue in equestrian sports [2,3,4,5,6,7,8,9,10,11,12]; however, scientific research on how to prevent these injuries is limited. The bit is a tool commonly used in ridden horses, yet the impact of its use on the horse’s well-being has only recently been questioned [2,3]. It has been shown that bits can cause severe lesions in the mouth, with lesions encountered in the mouth’s corners, the bars, and the tongue [4,5,6,7,8,9,10,11,12]. Scientific research is needed to optimize communication between horse and rider. Therefore, it is important to understand the dynamics of the bit in the horse’s mouth.

Fluoroscopic and radiographic studies have been used to observe the position and movements of different bit types within the oral cavity [13,14,15,16]. When applying bilateral rein tension, the joint of a snaffle bit pointed toward the incisor teeth, the bit rings moved caudally, and the tongue was indented (a nutcracker effect was not observed) [14,15]. The unilateral use of a rein caused the bit on the ipsilateral side to move caudally, while the contralateral side was pulled medially (inward movement) [13]. With a double-jointed snaffle, the entire mouthpiece moved toward the mandible, compressing the lingual tissues as it moved away from the palate [15]. Less intra-oral movement was observed in bits with an unjointed mouthpiece [14]. Although these studies provide valuable insights into the general action of the bit in the mouth, there is a lack of research on the factors that might influence the intra-oral movement of the bit.

The objective of this study is to examine the influence of bit type, bit size, rein tension, and rein angle on the movement of the bit.

## 2. Materials and Methods

**Figure 1:**
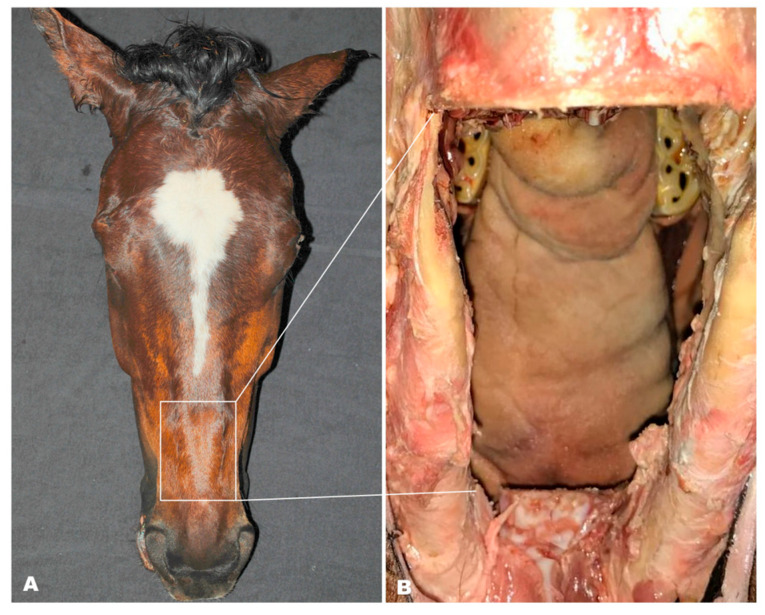
Illustration of the preparation of the cadaver heads to provide a dorsal window inside the mouth. Dorsal view of a cadaver head demonstrating the area (**A**) where a viewing window (**B**) is created after removal of skin, nasal bones, the nasal process of the left and right incisive bone, the nasal conchae, and the hard palate.

Five cadaver heads were obtained from horses euthanized for pathologies unrelated to the head. All were normal-sized, adult (age 5–15) warmblood horses with an intact dentition (all incisors and cheek teeth present). The heads were fixed on a custom-made table that allowed measurements with the reins at different angles and varying rein tension. A window was created on the dorsal aspect above the interdental space to allow observation of the bit and its movement from a dorsal perspective while keeping the maxillary bone and soft tissues on the lateral side (including the corners of the mouth) intact. To create this window, the skin, nasal bone, the caudodorsal part of the nasal process of the left and right incisive bones, and hard palate were removed (Figure 1).

A bridle (single bridle without noseband) was fitted to each head, and the bit was positioned in a standardized way with its caudal side at one-third of the distance between the mesial side of the mandibular 06 and the distal side of the 03. For each horse skull, the appropriate bit size was recorded. To provide a standardized assessment of the appropriate length of the bit for each cadaver head, a procedure was performed involving the application of a lateral force of 1 kg on the bit with a commercial spring scale device (Digital suitcase scale up to 50 kg. https://www.bol.com/nl/p/digitale-kofferweegschaal-tot-50kg-roestvrij-staal-grijs-bagage-weegschaal/9200000058382569/ accessed on 1 September 2025). The distance from the outside of the commissure to the inside of the bit rings was measured. The authors considered a distance of ±10 mm between the commissures and the bit as an appropriately fitted bit. The bits included in this study were snaffle bits (single/double-jointed; ring/eggbutt) and a curb bit. All bits were 16 mm thick. Eggbutt snaffle bits were used in three different sizes (11.5 cm, 12.5 cm, and 13.5 cm in length). The ring snaffle was 12.5 cm in length, and it was also recorded for each cadaver head whether this was an appropriately fitted bit. The curb bit was 12 cm in length. When using the curb bit, the chain strap was adjusted to ensure that the lever arms were positioned at an angle of approximately 45° relative to the line of the lips when rein tension was applied. Additionally, the width of the mandible was measured (distance between the left and right 06 at its mesial side).

**Figure 2:**
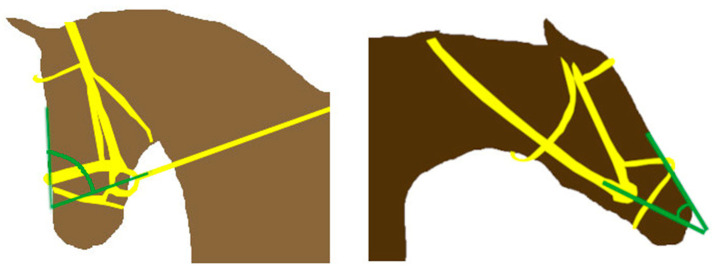
Illustration of a horse with the reins at a 70° angle to the long axis of the head, representing a flexed (poll flexion) head position on the left (e.g., during dressage riding). On the right, the illustration shows a horse with the reins at a 20° angle, representing an extended head and neck position (e.g., a horse during racing) (Angles shown in green).

Positional bit changes were recorded while exerting unilateral (left, right) rein tension and bilaterally symmetrical rein tension. Different degrees of rein tension (1, 2, and 4 kg) were applied in accordance with a study of measured rein tension during riding [17]. Measurements were performed with the reins at a 70° angle to the long axis of the head, which represents a flexed (poll flexion) head position, and repeated with the reins positioned at a 20° angle, which represents an extended head/neck position (Figure 2). All measurements were performed by the same person.

**Figure 3:**
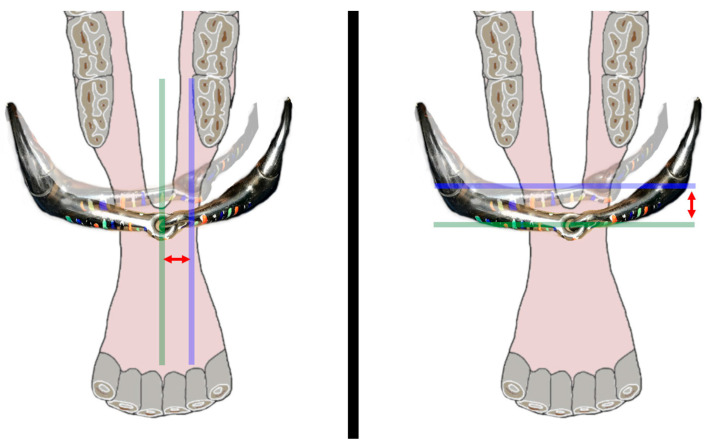
Schematic illustration of measured bit movements. A single-jointed eggbutt bit is shown in the neutral position and in transparent under unilateral tension to the left (right in the figure). On the left, a green sagittal line marks the central axis in neutral position, and a parallel purple line shows the bit’s position after rein tension. The distance between these lines indicates the lateral movement. On the right, a green horizontal line represents the neutral central axis, and a purple line marks the position after rein tension. The distance between these lines indicates posterior movement.

**Figure 4:**
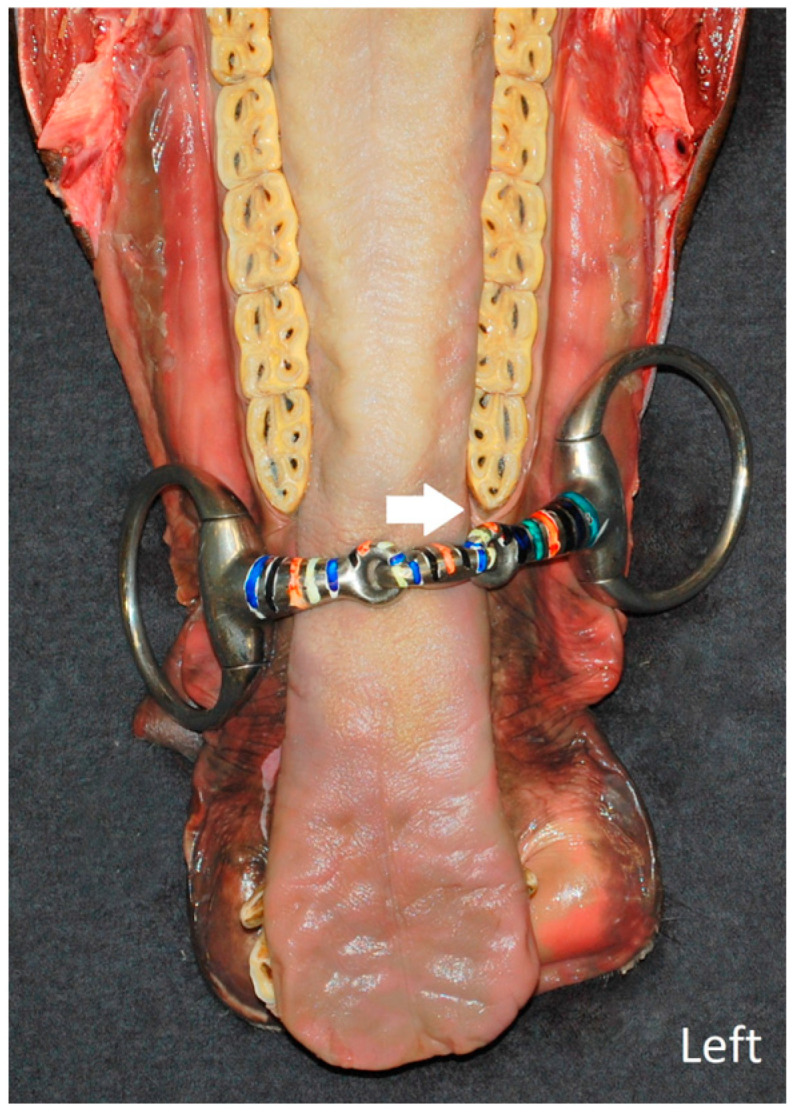
Dorsal view of a mandible in a cadaver head demonstrating the contact of the bit (double-jointed eggbutt snaffle, 12.5 cm) with the mesial gingival tissues of the mandibular 06s (white arrow).

Measurement of the degree of lateral and posterior movement of the bit was facilitated by the use of a custom-made transparent ruler that was fixed along the head’s midline, dorsal to the created viewing window, and by marking the bit with parallel-colored stripes every 5 mm. Positional changes in the center of the bit from the starting (midline) position were recorded (Figure 3). It was also noted if the bit made contact with the gingival tissues mesial to the mandibular 06 (Figure 4).

### Data Analysis

Statistical analysis was performed using commercial software (IMB Corp. IBM SPSS Statistics for Windows, Version 20.0., Armonk, NY, USA) (IBM SPSS 29). A linear mixed model was used to test the influence of bit ring type (ring/eggbut), joint (single/double), bit size (too small/fitting/too wide), angulation of the reins (20°/70°) and rein tension (1 kg/2 kg/4 kg), rein tension side (left/right/bilateral) on lateral and posterior displacement of the snaffle bit. A random effect of cadaver head was included in the model to account for dependent observations. Possible interactions between independent variables were explored using interaction plots and included in the model if interaction was present. Influences on displacement of curb-type bits were tested in a separate analysis using the same approach as for snaffle-type bits, but with only rein tension, angle, and rein tension side as independent variables, because the other independent variables were constant. For all models, model assumptions were evaluated on residual QQ plots and on residuals versus fitted values plots. When heteroscedasticity was detected, a logarithmic transformation of the dependent variable was carried out, which solved the issue. To test the effect of the independent variables on contact of the bit with the mandibular 06, a generalized linear mixed model with a binomial error distribution was used, including cadaver head as a subject effect. Also for this model, possible interactions were evaluated on interaction plots and included in the model if present. During model building, the model fit was evaluated using the Bayesian information criterion.

## 3. Results

In these five cadaver heads, the length of a properly fitting bit was 11.5 cm in three horses and 12.5 cm in two horses. The average width of the mandible of these 5 heads was 52.6 mm (±6.99 mm).

### 3.1. Snaffle Bit

#### 3.1.1. Lateral Movement of the Bit

A mean lateral bit displacement of 11.71 (±10.97) mm, 8.95 (±10.56) mm, 8.54 (±8.25) mm, and 9.09 (±9.58) mm was recorded for a single-jointed ring snaffle, a single-jointed eggbutt snaffle, a double-jointed ring snaffle, and a double-jointed eggbutt snaffle, respectively. A single-jointed ring snaffle displaced significantly more than a single-jointed eggbutt snaffle (*p* < 0.01). There was no significant difference between a double-jointed eggbutt and a ring snaffle bit (*p* = 1.06). A single-jointed ring snaffle displaced significantly more compared to a double-jointed ring snaffle (*p* < 0.01). No significant differences were found between a single-jointed or double-jointed eggbutt snaffle (*p* = 0.74).

The influence of different rein tensions on mean lateral displacement of the bit is illustrated in Table 1. Increasing unilateral rein tension significantly increases lateral bit displacement (*p* < 0.01), in contrast to symmetrical, bilaterally exerted forces (*p* > 0.99). The impact of rein angle on mean displacement of the bit is shown in Table 2. Statistical analysis showed that at the highest rein tension (4 kg), the bit displaced more significantly when the rein angle was 20° compared to 70° (*p* < 0.01). Mean lateral displacement of a properly fitting snaffle bit and a bit that is too wide is displayed in Table 3. With bilateral rein tension, there is no significant difference in displacement between bit sizes (*p* = 0.97). A bit that was considered too wide displaced significantly more when unilateral rein tension was exerted, compared to a properly fitting bit (*p* < 0.01).

#### 3.1.2. Posterior Movement of the Bit

A mean posterior bit displacement of 6.26 (±4.23) mm, 6.27 (±4.98) mm, 5.81 (±4.64) mm, and 5.52 (±5.13) mm was recorded for a single-jointed ring snaffle, a single-jointed eggbutt snaffle, a double-jointed ring snaffle, and a double-jointed eggbutt snaffle, respectively. No significant difference in posterior movement between a ring and eggbutt snaffle was observed (*p* = 0.83). In both single-jointed (*p* = 0.01) and double-jointed (*p* < 0.01) bits, significantly more posterior displacement was recorded with a bit that was considered too wide compared to a properly fitting bit. There was more posterior movement of the bit with a fitting single-jointed bit compared to a fitting double-jointed bit (*p* < 0.01). There was no significant difference in posterior movement between single-jointed and double-jointed bits that were too wide (*p* = 0.2).

The mean posterior displacements of the bit with different rein tensions are displayed in Table 1. Posterior displacement of the bit increased with increasing unilateral and bilateral rein tension (*p* < 0.01). The mean posterior displacements of the bit with the reins at an angle of 20° and 70° are displayed in Table 2. The posterior displacement of the bit also increased with increasing rein tension in both angles (*p* < 0.01). With a rein tension of 2 and 4 kg, there was significantly more posterior displacement of the bit with the reins at an angle of 20° compared to an angle of 70° (*p* = 0.01 and *p* < 0.01, respectively).

Bits that were considered too wide had higher odds of touching the gingival tissues mesial to the mandibular 06s compared to bits that were fitted properly (OR = 6.33, 95% CI = 2.13–18.84, *p* < 0.01). When the reins were angulated at 20°, the odds of contact between the bit and this area were higher compared to an angle of 70° (OR = 5.13, 95% CI = 2.47–10.68, *p* < 0.01). Higher rein tension (4 kg) also increased the odds of contacting the gingival tissues mesial to the mandibular 06s compared to a rein tension of 2 kg (OR = 46.11, 95% CI = 13.75–154.62, *p* < 0.01). With a rein tension of 1 kg, the bit never touched this region. Left- or right-sided differences were not noted.

Single-jointed ring snaffles contacted the 06 gingiva significantly more frequently than single-jointed eggbutt snaffles (OR = 6.04, 95% CI = 1.87–19.49, *p* < 0.01), which was not observed for double-jointed ring or eggbutt snaffles (OR = 1.65, 95% CI = 0.59–4.67, *p* = 0.34). A double-jointed eggbutt snaffle had higher odds of touching the 06 gingiva compared to a single-jointed eggbutt snaffle (OR = 22.24, 95% CI = 8.25–59.98, *p* < 0.01), in contrast to a single-jointed versus a double-jointed ring snaffle (OR = 2.24, 95% CI = 0.64–7.85, *p* = 0.21).

### 3.2. Curb Bit

Recorded mean lateral movement of the curb bit with a rein force of 1, 2, and 4 kg was 3.20 (±3.15) mm, 6.03 (±5.22) mm, and 10.10 (±8.14) mm, respectively. Lateral displacement was significantly higher with a unilateral rein force of 4 kg compared to 2 kg (*p* < 0.01) and 1 kg (*p* < 0.01), and with a unilateral rein force of 2 kg compared to 1 kg (left *p* = 0.02, right *p* < 0.01). The mean lateral movement with the reins at an angle of 20° and 70° was 6.33 (±6.22) mm and 6.56 (±6.77) mm, respectively, which did not differ significantly (*p* = 0.6).

The mean posterior movement of the bit with a rein force of 1, 2, and 4 kg was 0.60 (±0.62) mm, 2.07 (±1.66) mm, and 4.13 (±2.37) mm, respectively. Posterior displacement increased significantly with a bilateral rein force of 4 kg compared to 1 kg (*p* = 0.01). Posterior movement of the bit significantly increased with a unilateral rein tension of 4 kg compared to 2 kg (*p* < 0.01) and 1 kg (*p* < 0.01); and with 2 kg compared to 1 kg (left *p* = 0.01, right *p* = 0.02). There was significantly higher posterior movement of the curb bit with the reins at an angle of 20° (mean: 2.78 ± 2.22 mm) compared to 70° (mean: 1.76 ± 2.16 mm) (*p* < 0.01). The influence of different rein tensions and rein angles on mean displacements of the curb bit is illustrated in Table 4 and Table 5.

Contact with the mesial gingival margin of the mandibular 06 was never observed.

## 4. Discussion

This study provides valuable preliminary ex vivo information about the potential positional changes in the bit in the mouth under different settings. It demonstrates that bit design and dimensions influence the movement of the bit within the horse’s mouth in an ex vivo setting. Furthermore, it shows that the horse’s head position and the rider’s tension on the reins affect this movement.

Literature on the freedom of movement of the bit inside the mouth is scarce [13,14,18], and the influence of this movement on oral lesions remains unknown. Based on personal observations, the authors hypothesized that bit-induced oral lesions are more likely to occur when the bit is too wide. This might be due to an increased ability of the bit to move inside the mouth, potentially entrapping soft tissues more easily. However, further research is necessary to confirm this hypothesis.

Bits function by moving laterally and/or posteriorly when pressure is applied to the reins [19]. The use of the bit is intended to apply pressure to various intra-oral structures (e.g., the lips, diastema, hard palate, and tongue) and can be a potential source of discomfort for the horse, as the mouth is a highly sensitive area [20,21]. Bit-induced lesions are reported to occur frequently [8,9,10,12], but the interaction between the bit and the oral structures is a complex, multifactorial process, making it difficult to draw straightforward conclusions regarding the direct causes of these lesions. There is a clear need for more scientific research to better understand the dynamics of the bit in the mouth and its potential consequences for horse welfare. Part of the required information is identifying which factors influence these dynamics. Previous studies have identified factors such as the rider (e.g., rein pressure), the horse (e.g., head position), and the bit itself as influencing intra-oral dynamics [13,14,17,19].

A limitation of this study stems from its cadaveric model, which inherently precludes dynamic interactions with soft oral tissues such as tongue, lips, and cheeks. In a live horse, the tongue is an active muscle, often retracting or bulging in response to rein tension, which are actions that influence bit positioning [13,14,15,16]. Additionally, our study focused on lateral and posterior movements of the bit within the oral cavity. However, the bit is capable of other complex motions, which can significantly impact contact with oral structures, depending on bit design and rein tension. Moreover, the influence of ancillary equipment (such as nosebands, bridle fit, and curb chains) on bit dynamics was not considered. Despite these limitations, our cadaver model remains a valuable baseline tool. It allows controlled evaluation of how variations in bit characteristics can impact movement within the confines of the oral cavity.

Another important limitation of this study is the relatively small sample size of cadaver heads tested. This inherently restricts the statistical power of the analysis and reduces the generalizability of the findings. With limited numbers, subtle but potentially relevant differences between bit types or sizes may not have been detected, while observed effects should be interpreted with caution. Consequently, the outcomes presented here should be regarded as potential effects rather than definitive conclusions, serving primarily as an indication of possible trends that warrant confirmation in larger-scale or in vivo studies.

This study demonstrated that increasing rein tension significantly increases the degree of both lateral and posterior movement of the bit. The relationship between rein tension and the pressure exerted on oral structures is not fully understood, but it has been suggested that the risk of bit-related lesions increases with higher rein tension [19,22]. Rein tension is influenced by various factors, including the horse, the rider, and the training equipment used [23]. While it is generally accepted that maintaining the lightest possible contact during riding (about 400 g) is desirable [24], studies have shown that the rider’s perception of rein tension does not always correlate with objective measurements [25,26]. Therefore, objective measurement of rein tension seems to be crucial to achieving this lightness, and rein tension devices could be used during both training and competition to ensure appropriate pressure levels [24].

Head and neck positions also influence bit dynamics, with a more stretched head-neck relationship leading to greater displacement, especially under higher rein tension. Bennet has suggested that lesions are more likely to occur when the nose is extended [19]. Therefore, the authors recommend that riders exercise extra caution when applying rein tension in this position.

There are various types of bits used in equine sports, each differing in design, length, and diameter. However, little scientific information is available on how these variations affect the dynamics inside the mouth. Previous studies have provided some basic insights into the interaction between the bit and the oral cavity [13,14,15,16]. Our findings support those of Clayton and Lee, who observed that bits that are too wide (or positioned too low) in the mouth exhibit an increased range of movement [13]. A properly fitted bit appears to remain more stable in the horse’s mouth, which may reduce the likelihood of inducing traumatic lesions. However, further research is needed to establish a definitive link between bit stability and lesion prevention.

When fitting a bit, the anatomical structures of the rostral aspect of the horse’s mouth must be considered. Engelke and Gasse suggested that the thickness of the bit should be based on the distance between the upper and lower jaws at the level of the interdental space. While mandibular width was not studied by these authors, the present study, which used a limited sample of normal-sized horse heads, found that the mean measured mandibular width (52.6 ± 6.99 mm) is incompatible with the use of overly wide bits (e.g., 140 mm). Manfredi et al. fitted the length of the bit by ensuring the mouthpieces were either the same width or up to 0.5 cm wider than the distance between the left and right commissures of the lips [5]. The authors of this study have developed a standardized method to determine bit length, considering a margin of ±1 cm as a properly fitted bit.

Curb bits are leverage bits, meaning that the tension applied by the rider is amplified by the bit [1]. Our study confirms Clayton’s observation that the overall intra-oral movement is much more limited with curb bits compared to snaffle bits [14], although both types of bits are influenced by the same parameters. Lesions at the level of the bars and tongue are specifically associated with curb bits [9], suggesting that curb bits are unlikely to cause lesions through movement alone. Instead, the primary factor in inducing bit lesions is the localized pressure exerted by the bit on these areas. This supports the conventional advice against using curb bits on untrained horses or by inexperienced riders, as untrained riders tend to apply higher rein tension [24].

Tuomola et al. emphasized the importance of carefully examining and palpating the area near the second lower premolar for lesions, as these lesions are difficult to detect [12]. To cause such lesions, the bit must be able to contact this region. This study showed that bits that are fitted too wide, with reins at a 20° angle and a rein tension of 4 kg, are significantly more likely to contact the mesial gingival tissues of the mandibular 06s. This finding suggests that these factors—wider bits, greater rein tension, and a more extended head position—may contribute to lesions in this specific area. Since no contact with the mesial gingival tissues of the mandibular 06s was observed with the curb bit, it can be inferred that lesions in this zone are more typical for snaffle bits. Specifically, a single-jointed ring snaffle made significantly more contact with the 06s compared to a single-jointed eggbutt snaffle. However, a double-jointed eggbutt snaffle had a higher likelihood of making contact with the 06s compared to a single-jointed eggbutt snaffle. These findings suggest that a properly fitted single-jointed eggbutt snaffle may be the least likely of the examined snaffle bits to cause contact with this area, and may therefore be less likely to induce lesions in the mesial gingival margin of the mandibular 06s.

Due to the large variety of bit designs available and given that this is the first study of its kind, the authors chose to limit the number of bit types included. It would be interesting to examine a wider range of bit designs and sizes, as some factors may influence the movement of other bits differently. This cadaver model also provides an understanding that can inform and guide future in vivo investigations that incorporate the complexity of equestrian tack and oral biomechanics.

## 5. Conclusions

Although this ex vivo setting does not fully replicate natural conditions, it provided valuable insight into the potential effects of bit characteristics on intra-oral movement. This study demonstrated that bit design, bit size, the horse’s head position, and rein tension can influence the movement of the bit within the mouth. Increased rein tension results in more movement of the bit, both laterally and posteriorly, and this movement is amplified when the horse adopts a more extended head-neck position. Additionally, an oversized bit exhibits more movement within the mouth. The authors hypothesize that increased bit movement may heighten the risk of soft tissue entrapment, potentially leading to lesions. By controlling factors that promote excessive movement of the bit, the likelihood of bit-related trauma may be reduced. However, further research is required to substantiate this hypothesis.

## Figures and Tables

**Table 1:** Positional changes in the snaffle bit (in mm + standard deviation) following exertion of a rein tension of 1, 2, and 4 kg, respectively.

Rein Tension	1 kg	2 kg	4 kg
Lateral movement left	6.79 (±4.14)	12.44 (±5.73)	21.61 (±7.62)
Lateral movement right	6.39 (±3.47)	13.60 (±6.45)	22.31 (±9.43)
Lateral movement bilateral	0.06 (±0.56)	0.2 (±1.18)	0.26 (±1.71)
Posterior movement left	1.43 (±1.38)	4.72 (±2.13)	10.59 (±4.98)
Posterior movement right	1.61 (±1.22)	5.29 (±3.14)	10.69 (±5.42)
Posterior movement bilateral	2.65 (±1.58)	6.03 (±2.61)	10.36 (±4.93)

**Table 2:** Positional changes in the snaffle bit (in mm + standard deviation) with rein tension exerted at an angle of 20° or 70° to the long axis of the horse’s head.

Rein Tension	1 kg	2 kg	4 kg
Lateral movement 20°	4.30 (±4.37)	8.88 (±8.14)	15.87 (±13.27)
Lateral movement 70°	4.53 (±4.43)	8.62 (±7.63)	13.58 (±11.49)
Posterior movement 20°	1.92 (±1.48)	5.67 (±2.53)	11.78 (±5.49)
Posterior movement 70°	1.88 (±1.52)	5.03 (±2.84)	9.31 (±4.35)

**Table 3:** Lateral movement of a snaffle bit that is properly fitting or too wide (mm + standard deviation) influenced by unilateral or bilateral rein tension.

Rein Tension	Fitting Bit	Too Wide
Bilateral	0.04 (±0.24)	0.3 (±1.66)
Left	11.87 (±8.18)	15.51 (±8.68)
Right	11.82 (±7.92)	16.51 (±10.11)

**Table 4:** Positional changes in the curb bit (in mm + standard deviation) following exertion of a rein tension of 1, 2, and 4 kg, respectively.

Rein Tension	1 kg	2 kg	4 kg
Lateral movement left	5.20 (±1.64)	9.00 (±3.40)	16.30 (±3.30)
Lateral movement right	4.40 (±2.50)	8.70 (±3.16)	13.9 (±5.08)
Lateral movement bilateral	1.10 (±1.98)	2.3 (±3.68)	0.3 (±0.54)
Posterior movement left	0.80 (±0.48)	2.80 (±1.20)	5.70 (±1.78)
Posterior movement right	0.40 (±0.48)	1.70 (±0.70)	4.20 (±1.40)
Posterior movement bilateral	0.80 (±0.80)	2.00 (±1.80)	2.00 (±1.00)

**Table 5:** Positional changes in the curb bit (in mm + standard deviation) with rein tension exerted at an angle of 20° or 70° to the long axis of the horse’s head.

Rein Tension	1 kg	2 kg	4 kg
Lateral movement 20°	2.80 (±2.45)	6.67 (±4.75)	9.53 (±6.82)
Lateral movement 70°	3.60 (±2.91)	5.40 (±4.43)	10.67 (±7.87)
Posterior movement 20°	0.93 (±0.37)	2.80 (±1.39)	4.60 (±1.41)
Posterior movement 70°	0.27 (±0.39)	1.33 (±0.71)	3.67 (±2.09)

## Data Availability

The original contributions presented in this study are included in the article. Further inquiries can be directed to the corresponding author(s).
